# Information Technology–Based Management of Clinically Healthy COVID-19 Patients: Lessons From a Living and Treatment Support Center Operated by Seoul National University Hospital

**DOI:** 10.2196/19938

**Published:** 2020-06-12

**Authors:** Ye Seul Bae, Kyung Hwan Kim, Sae Won Choi, Taehoon Ko, Chang Wook Jeong, BeLong Cho, Min Sun Kim, EunKyo Kang

**Affiliations:** 1 Office of Hospital Information Seoul National University Hospital Seoul Republic of Korea; 2 Department of Family Medicine Seoul National University Hospital Seoul Republic of Korea; 3 Department of Thoracic and Cardiovascular Surgery Seoul National University Hospital Seoul Republic of Korea; 4 Department of Thoracic and Cardiovascular Surgery Seoul National University College of Medicine Seoul Republic of Korea; 5 Department of Emergency Medicine Seoul National University Hospital Seoul Republic of Korea; 6 Department of Urology Seoul National University Hospital Seoul Republic of Korea; 7 Department of Public Health and Medical Service Seoul National University Hospital Seoul Republic of Korea; 8 Department of Pediatrics Seoul National University Hospital Seoul Republic of Korea

**Keywords:** COVID-19, clinical informatics, mobile app, telemedicine, hospital information system, app, health information technology

## Abstract

**Background:**

South Korea took preemptive action against coronavirus disease (COVID-19) by implementing extensive testing, thorough epidemiological investigation, strict social distancing, and rapid treatment of patients according to disease severity. The Korean government entrusted large-scale hospitals with the operation of living and treatment support centers (LTSCs) for the management for clinically healthy COVID-19 patients.

**Objective:**

The aim of this paper is to introduce our experience implementing information and communications technology (ICT)-based remote patient management systems at a COVID-19 LTSC.

**Methods:**

We adopted new electronic health record templates, hospital information system (HIS) dashboards, cloud-based medical image sharing, a mobile app, and smart vital sign monitoring devices.

**Results:**

Enhancements were made to the HIS to assist in the workflow and care of patients in the LTSC. A dashboard was created for the medical staff to view the vital signs and symptoms of all patients. Patients used a mobile app to consult with their physician or nurse, answer questionnaires, and input self-measured vital signs; the results were uploaded to the hospital information system in real time. Cloud-based image sharing enabled interoperability between medical institutions. Korea’s strategy of aggressive mitigation has “flattened the curve” of the rate of infection. A multidisciplinary approach was integral to develop systems supporting patient care and management at the living and treatment support center as quickly as possible.

**Conclusions:**

Faced with a novel infectious disease, we describe the implementation and experience of applying an ICT-based patient management system in the LTSC affiliated with Seoul National University Hospital. ICT-based tools and applications are increasingly important in health care, and we hope that our experience will provide insight into future technology-based infectious disease responses.

## Introduction

The coronavirus disease (COVID-19) pandemic has become a major concern worldwide since the first report from Wuhan, China in December 2019 [1-4]. According to the World Health Organization, up to 4 million patients and 277,000 deaths have been reported [5,6]. In South Korea, the first confirmed case of COVID-19 was diagnosed on January 20, 2020; as of May 10, 2020, 10,874 patients have been diagnosed, 9610 have been released from isolation, and 256 have died [7].

South Korea has earned recognition worldwide for its tackling of the pandemic. The number of infections dropped from 900 a day in late February to less than 20 per day in late April. South Korea acted preemptively against COVID-19 with robust testing, vigorous tracing, strict social distancing, and rapid treatment of patients according to severity. In late February, a super-spreader, “Patient 31,” caused a regional outbreak in the southern city of Daegu and Gyeongsangbuk Province by attending religious services. Health authorities conducted contact tracing and decided to test every close contact irrespective of whether they showed any symptoms [8]. Up to 80% of patients tested were clinically healthy, and due to the substantial number of tests performed, medical institutions were highly saturated. To provide appropriate treatment and to reduce the burden on the medical institutions of Daegu and Gyeongsangbuk Province, the government established a new treatment system based on severity on March 1, 2020 [9]. Every patient was classified based on severity into mild, moderate, severe, or extremely severe cases. Patients identified as moderate, severe, and extremely severe were immediately hospitalized for treatment [9,10].

Asymptomatic patients testing positive for COVID-19 and mild symptomatic patients were isolated and accommodated at government-sponsored facilities called living and treatment support centers (LTSCs) to be monitored by health care staff at least twice a day [9]. Patients can be discharged from the LTSC after two consecutive negative tests. In case of emergencies, patients are transferred to nearby hospitals [9]. The government asked several tertiary hospitals to operate LTSCs. The first LTSC opened in Daegu; Seoul National University Hospital (SNUH) opened the third LTSC at the SNUH Human Resource Development Center in Mungyeong, Gyeongsangbuk Province, 180 kilometers southeast of Seoul and 100 kilometers northwest of Daegu.

In this study, we introduce the experience of operating a LTSC for the management of mild COVID-19 patients and introducing information and communications technology (ICT)–based solutions tailored to the characteristics of the patients’ clinical pathways.

## Methods

We implemented new electronic health record (EHR) templates for the hospital information system (HIS), dashboards, an electronic prescription (e-prescription) system, and cloud-based medical image sharing. A newly developed mobile app enabled effective and accurate communication between health care providers and patients, and wearable vital sign monitoring devices facilitated management of clinically healthy COVID-19 patients.

### The SNUH HIS

SNUH is a non-profit, academic referral government hospital with 1778 beds. SNUH treats approximately 600,000 inpatients and 2.3 million outpatients per year and employs about 1400 physicians and 2200 nurses. SNUH internally developed a computerized physician order entry system in 1999, implemented a picture archiving and communication system (PACS) in 2001, and started using an EMR in 2004. In 2001, SNUH’s internal information technology (IT) department was spun off as a separate company, ezCaretech, which is now the largest HIS company in Korea [11]. The current version of the HIS, BESTCare 2.0, which integrates and manages all services needed by the hospital, was built by ezCaretech and implemented in 2016. The HIS was modified to support the LTSC.

### Patient Management Systems to Support a LTSC in Mungyeong

Health authorities screened and designated clinically healthy COVID-19 patients to be admitted to LTSCs; however, patient management was entirely the responsibility of the entrusted hospital. Patients were considered clinically healthy if their vital signs were stable (blood pressure, heart rate, and oxygen saturation within normal limits), if they were afebrile, and if they were asymptomatic or had mild COVID-19 symptoms. Clinical health status was determined by directly evaluating the patient at the time of diagnosis. Blood pressure monitors, pulse oximeters, and thermometers were placed in each room for the patient to measure their blood pressure, oxygen saturation, and temperature. In some rooms, a wearable continuous vital sign monitoring device was placed alongside conventional devices for continuous remote monitoring. To protect the medical staff, exposure was minimized via the use of remote communication methods and self-monitoring by the patients.

### User Survey

We conducted online surveys to measure satisfaction with the systems modified for the LTSCs. Three separate online surveys were conducted: patient satisfaction with the mobile app, patient satisfaction with the wearable device, and satisfaction of the medical staff. Only participants who voluntarily agreed to participate were included. Responses were measured using a 5-point Likert scale. The questionnaires were administered using an online survey form. Participants accessed questionnaires via a URL and were able to complete the survey at any time or place. The study was approved by the Institutional Review Board of SNUH (IRB number: H-2004-026-1115). The ethics committee waived the need for informed participant consent.

## Results

### Patient Management in the LTSC

Patients who were positive for the COVID-19 real time polymerase–chain reaction (RT-PCR) test but were asymptomatic or had mild symptoms were subject to LTSC admission. Upon disease confirmation, a group of experts from public authorities triaged the patient and determined treatment depending on the severity of the patient’s symptoms. Patients with severe symptoms were hospitalized and treated in negative pressure isolation units, and mild and asymptomatic patients were designated to be admitted to LTSCs [7-9]. Patients were initially admitted to the LTSC from self-isolation in their homes or were transferred from other local hospitals.

As of April 20, 2020, a total of 18 LTSCs were operational in Korea. 2948 patients were admitted to the centers, of whom 155 were transferred to another hospital due to worsening symptoms [12]. Because the number of newly diagnosed patients decreased and the number of people who were released from quarantine increased, the 18 LTSCs were consolidated into 5 centers in May 2020.

The SNUH facility in Mungyeong was originally constructed as a human resource development center used to train SNUH personnel. It was not a medical facility and therefore did not contain any medical equipment. After the decision was made to adapt the facility at Mungyeong into a LTSC, the necessary equipment, including personal computers, monitors, a portable chest x-ray machine, medical devices, and the hospital network, was installed in 2 days. Since the LTSC opened on March 5, 2020, a total of 118 patients have been admitted.

**Figure 1:**
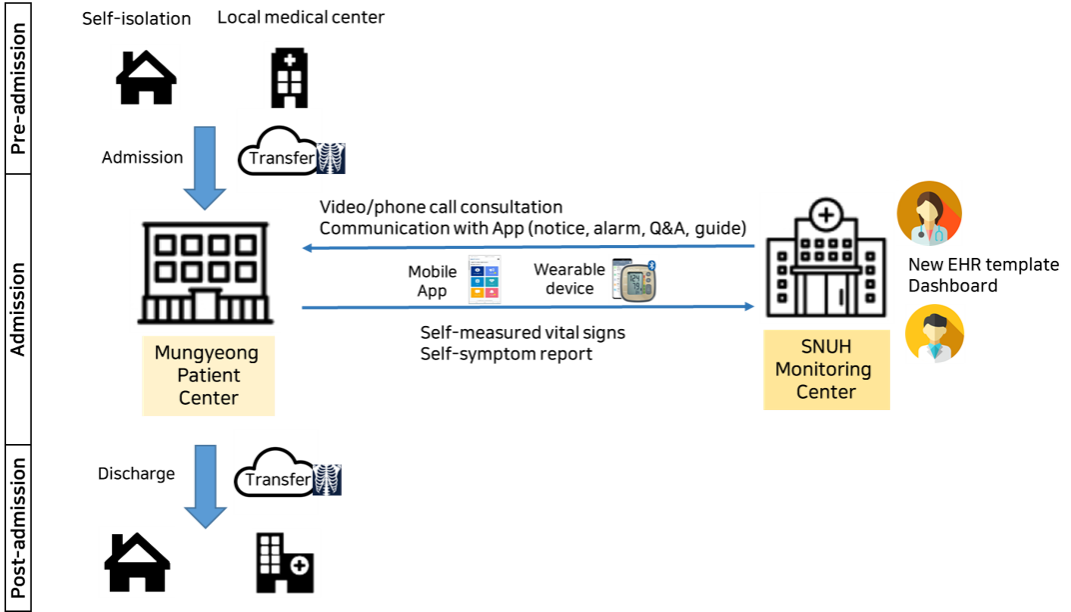
Overall ICT operation flow according to the patient's journey. EHR, electronic health record. ICT: information and communications technology. Q&A: question and answer. SNUH: Seoul National University Hospital.

Figure 1 shows the overall flow of ICT operations according to the patient journey. A cloud system was used for instant medical image sharing in cases of patient transfer. Patients self-reported vital signs and subjective symptoms through a mobile app. These data were automatically linked to the newly introduced semistructured EHR template for mild COVID-19 patients in the HIS to help the medical staff obtain patient-reported data conveniently and accurately. In addition, in some rooms equipped with wearable devices, vital signs were transmitted to the HIS in real time. Through the mobile app, medical staff were able to notify and alert individual patients and communicate with them when needed. The Korean government temporarily permitted virtual patient management to minimize COVID-19 transmission on February 22, 2020 [7]. Each patient self-measured vital signs and reported symptoms through the mobile app at 9:00 AM and 4:30 PM every day, and they spoke with the nurse in charge via remote video consult twice a day from 9 AM-12 PM and 5 PM-8 PM and once a day with a physician in Seoul. If the patient had a concern or problem, the assigned nurse could be contacted by text or telephone. To respond to emergencies, more than two medical staff members (physicians or nurses) were always present in the patient center at Mungyeong.

Patients were discharged if they had two consecutive negative COVID-19 tests. The RT-PCR test used by SNUH is the STANDARD M nCov Real-Time Detection Kit (SD Biosensor), which was issued an emergency use authorization by the United States Food and Drug Administration and the Korea Food and Drug Administration. The sensitivity of the test is 100% and the specificity is greater than 97% [13,14]. Chest x-rays were performed periodically to check for progression to pneumonia after admission [7-9].

The Korean government funded the operation of the LTSCs through reimbursement of medical fees. The conditions for reimbursement were as follows: medical personnel are dispatched to the LTSC, patients are seen twice a day, and necessary medical equipment, such as an x-ray machine, is available. The fee of about US $30 per patient per day could be claimed if the conditions were met [15].

### Enhancements to the HIS

A multidisciplinary task force of physicians, nurses, infection specialists, epidemiologists, and IT experts headed by the chief information officer of the hospital was convened to direct and manage the modifications to the IT systems to support the LTSC. First, we customized and enhanced our HIS to support the LTSC.

#### New EHR Templates and Order Codes

**Figure 2:**
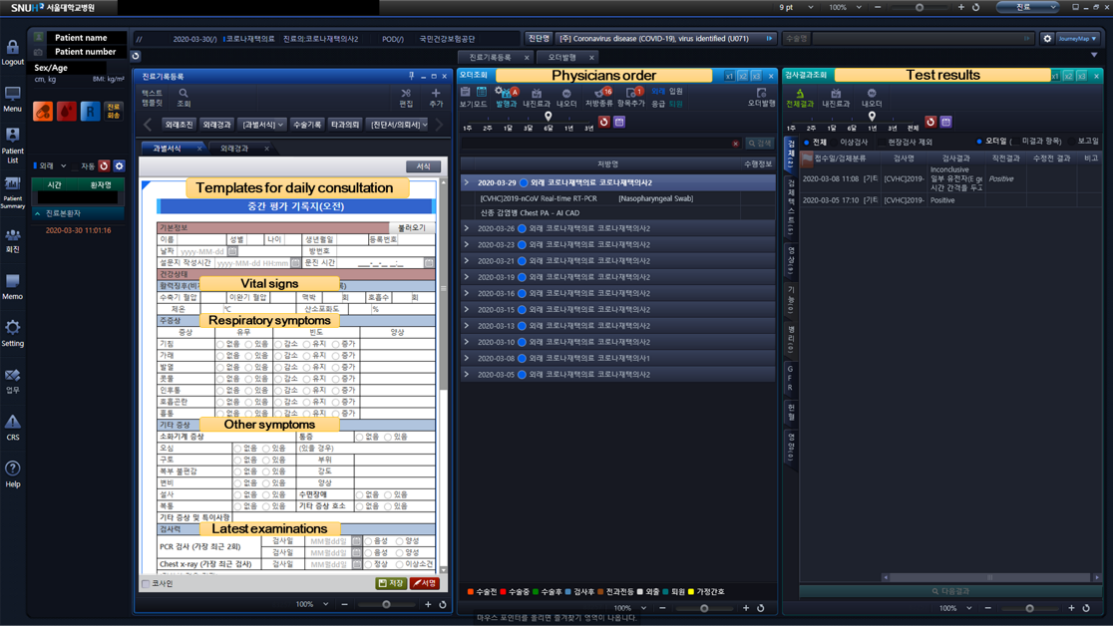
Modified EHR template for daily consultation at the LTSC.

To use the SNUH HIS in the LTSC, a hospital network was installed in addition to the usual internet service. An LTSC department was established in the HIS for efficient and independent patient management. Only chest x-rays and RT-PCR using a nasopharyngeal swab or sputum samples were performed at the LTSC; new order codes were created for these tests. Moreover, we developed three new EHR templates for admission, progression, and discharge, which were used to track the symptoms and general status of the patients (Figure 2). The EHR templates for the LTSCs were constructed in a semistructured form that combined a free text section for medical staff to freely record observations during consultation and a structured form that was configured to include patients’ subjective respiratory, gastrointestinal, and psychological symptoms, objective vital signs, previous medical history, contact history of COVID-19, and allergies. When a patient entered self-reported symptoms or vital signs on the mobile app, the data were automatically linked to the EHR template to be viewed during remote video consultation.

#### Dashboard

**Figure 3:**
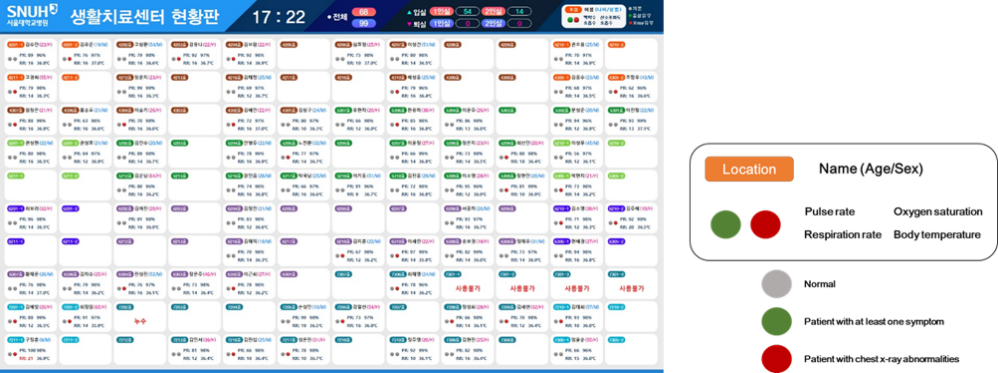
Dashboard of the EHR displaying real time information for patients at the LTSC.

A dashboard for the medical staff was developed to display real time information for all patients at central monitoring centers at Seoul and Mungyeong. The dashboard displayed each patient’s vital signs (heart rate, body temperature, respiratory rate, blood oxygen saturation) and information on whether the patient has any symptoms or chest x-ray abnormalities (Figure 3).

#### e-Prescription System

Basic medications were prepared at SNUH and transported to the LTSC facility. Plans were made for sourcing other medications from local pharmacies by sending prescriptions electronically. A mobile app–based e-prescription system was added to the SNUH app for patients on March 1, 2020. The patient could send the prescription information through the app to a nearby pharmacy selected by the user. There were two cases of e-prescription system usage in the LTSC. A 25-year-old female patient taking propranolol regularly for palpitation was admitted but did not bring the medication with her. During the stay, she complained of palpitations with tachycardia. The physician at the central monitoring center in Seoul prescribed propranolol, and the prescription was electronically sent through the app to a local pharmacy about 1.8 km from the LTSC. Another case was a 25-year-old male patient with hyperthyroidism who was prescribed and received methimazole using the e-prescription system.

### Cloud-Based Medical Image Sharing

**Figure 4:**
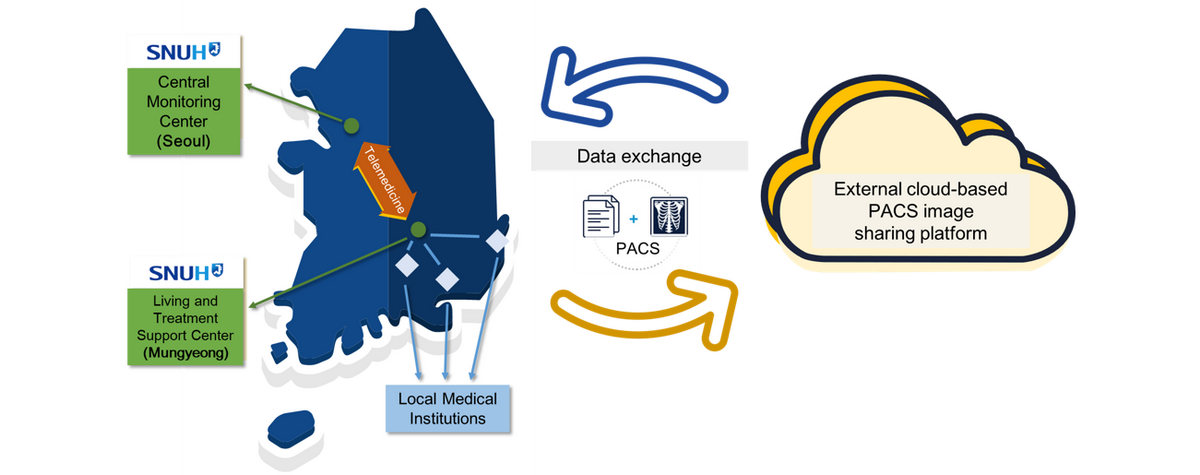
The cloud-based medical image sharing platform. PACS: picture archiving and communication system. SNUH: Seoul National University Hospital.

Seamless medical image interoperability is not available in Korea. Generally, medical images are copied to CDs and carried by the patient from one institution to another. Clearly, this process is inadvisable due to the nature of the disease. In addition, SNUH decided to review the chest x-ray and computerized tomography images taken by radiologists at SNUH before a to the LTSC. Also, producing a CD copy for patients in need of transfer to another hospital due to emergent or worsening conditions was not practical. Thus, we decided to establish a simple cloud-based system for instant and effective medical image sharing (Figure 4). Users from other hospitals would upload the patient’s medical images to the cloud, which were then transferred to the SNUH PACS for physician review. All medical images were shared using the Digital Imaging and Communications in Medicine (DICOM) format. Twelve patients used this system when they were admitted. Two patients who had no symptoms at the time of admission developed difficulty breathing and were transferred to a nearby hospital. Chest x-rays taken at LTSC were quickly shared with medical staff in the receiving hospital using this system.

### Wearable Vital Sign Monitoring Device

Some patients were asked to use a wearable device to measure their vital signs and enable the medical staff to monitor them remotely. The Vital-sign Data Recorder (VDR-1000) from Tribell Labs is a wearable medical multi-function measurement device that can be easily used by patients (Multimedia Appendix 1). It can directly measure the patient’s electrocardiogram, pulse rate, blood oxygen saturation, respiratory waveform, respiratory rate, and body temperature. The measured data are transmitted to the central monitoring system (CMS) using Wi-Fi and are then forwarded to the HIS. Medical staff in Mungyeong and Seoul can monitor vital signs of patients using both the CMS monitor and HIS (Multimedia Appendix 2). The CMS software can set alarms with different thresholds for each patient. An alarm sounds when a value outside the threshold range is measured, assisting the medical staff to respond quickly (Multimedia Appendix 3).

### Mobile App

**Figure 5:**
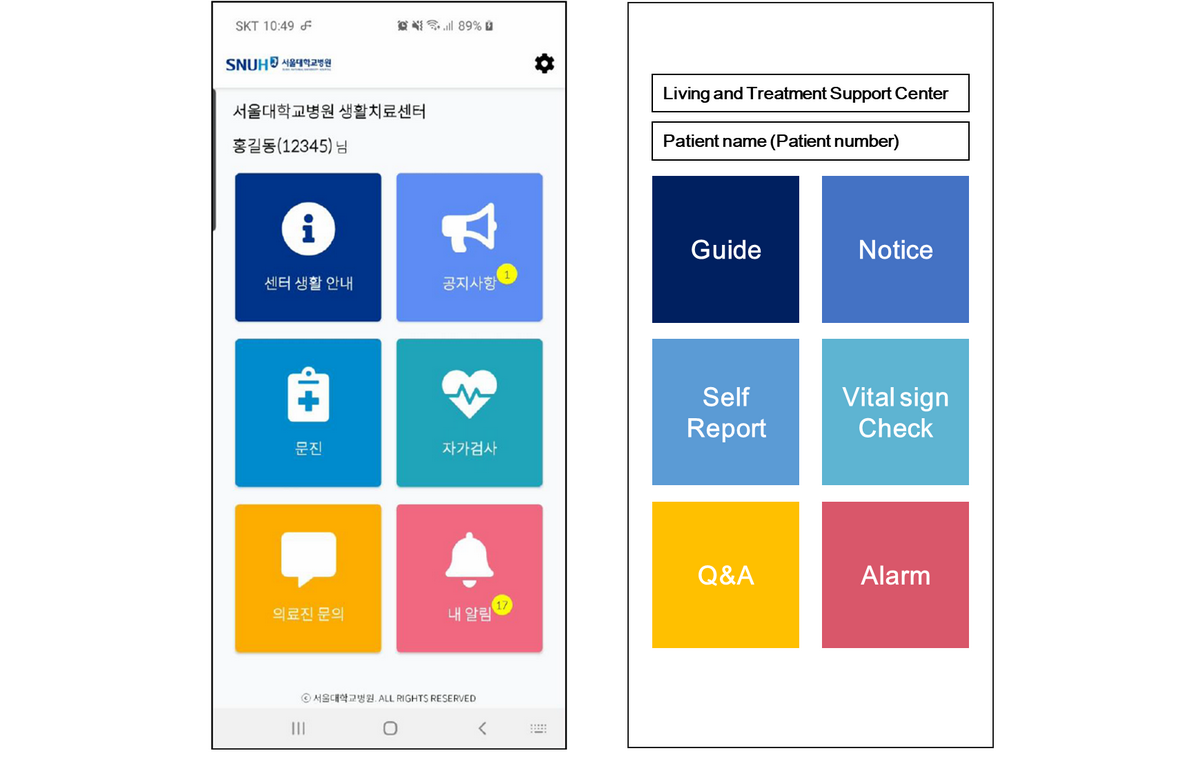
Interface of the mobile app for patients admitted to the LTCS. Q&A: question and answer.

To enable efficient patient management and communication between the patients and medical staff, an Android-based mobile app was developed for patients in the LTSC. The app consisted of six functions: general guides for patients admitted to the LTSC, notice board, symptom questionnaire, vital sign reporting, question and answer (Q&A), and push reminders. The app was released on March 27, 2020 (Figure 5).

#### General Guides for the LTSC

General information on admission to the LTSC and frequently asked questions by patients were organized for patient reference. Administrators could edit or register additional content directly through the app management web portal.

#### Notice Board

The notice board was used to send announcements to the patients. A push alarm was triggered when a new announcement was registered.

#### Symptom Questionnaire

Patients were asked to self-report their symptoms using the questionnaire in the mobile app. The questionnaire focused on the presence and frequency of symptoms most relevant to COVID-19 (cough, sputum, fever, rhinorrhea, sore throat, dyspnea, and chest pain) but also asked patients to report other more general symptoms (nausea, vomiting, abdominal discomfort, constipation, diarrhea, abdominal pain, insomnia, pain, and others). The self-reported data automatically populated the health record template.

#### Vital Sign Self-Reporting

In addition to the self-reporting of symptoms, patients were instructed to measure their vital signs twice a day, in the morning and afternoon, with the devices provided in each room and to enter the values into the mobile app. The vital sign measurements were also sent to the HIS to be reviewed by the medical staff.

#### Q&A

The Q&A function was a secure messaging platform for patients to submit questions to the medical team. The staff members replied through the app management web portal, and when a question was answered, a push notification was sent to the patient’s mobile phone.

#### Push Alarms

Patients received a push alarm as a reminder to fill out the self-reporting questionnaires for symptoms and vital signs, when their question was answered, or when the medical staff posted a new announcement to the notice board.

### Usability Survey

Twelve patients participated in the online surveys. The mean age of the respondents was 25 years (SD 6.25 years), and 7 (58%) were college students. For the mobile app, out of 5 points, the perceived usefulness showed the highest score at 4.62 points (SD 0.48), followed by satisfaction at 4.08 points (SD 1.41) and perceived ease of use at 3.81 points (SD 0.41) (Supplemental Table 1 in Multimedia Appendix 4). When patients rated the experience of using the wearable vital sign monitoring devices out of 5, perceived usefulness scored the highest at 4.45 points (SD 0.57), followed by perceived ease of use at 4.30 points (SD 0.59) and satisfaction at 3.98 points (SD 0.70) (Supplemental Table 2 in Multimedia Appendix 4).

A simple satisfaction survey regarding the usability of the mobile app was also conducted with the medical staff. Of the 24 participants who answered the questionnaire, 20 (83%) were nurses and 4 (17%) were physicians. The overall satisfaction score for the mobile app out of five points was 4.10 (SD 0.64) (Supplemental Table 1 in Multimedia Appendix 4).

## Discussion

### Principal Findings

The ICT-based patient management system introduced in this study was applied to the LTSC for clinically healthy COVID-19 patients operated by SNUH. Due to the infectious nature of the disease, it was difficult for medical staff to directly perform patient care; therefore, noncontact management was essential. SNUH introduced an ICT-based care system for the management of clinically healthy COVID-19 patients at the LTSC that fit the entire care flow, from preadmission and admission to discharge or transfer. The process started on March 1, 2020, and was completed in 4 weeks. Korea’s strategy of aggressive mitigation through early detection based on extensive diagnostic testing thorough epidemiological investigation and transparent management of COVID-19–related information has “flattened the curve” of the rate of infection [7]. The Ministry of Health and Welfare of Korea has tailored treatment policies and guidelines to match the disease profile of COVID-19 and the available medical resources. As part of their multifaceted approach to COVID-19, the Ministry relaxed regulations and temporarily approved the limited use of telemedicine and e-prescriptions [9]. Because direct telemedicine visits between health care professionals and patients were previously prohibited, the infrastructure and ecosystem around telemedicine were not fully developed [16]. Another approach was the decision to isolate patients with mild symptoms in designated LTSCs from March 1, 2020 [9]. The LTSCs were public or private facilities that were modified to accommodate and quarantine patients. SNUH, as a national hospital of Korea, was also asked to operate an LTSC at the SNUH Human Resources Development Center in Mungyeong. Most of the medical staff were located in the central monitoring center in Seoul and led patient care using telemedicine tools; meanwhile, some staff worked at the Mungyeong site in supportive roles and in preparation for emergencies.

The authors recognized the importance of a multidisciplinary approach to develop a system to support patient care and management at LTSCs as quickly as possible [17-19]. The Office of Hospital Information of SNUH, composed of several medical faculty members, nurses, pharmacists, medical record technicians, and IT experts, manages the IT services of the hospital and was well placed to lead this effort. Moreover, ezCaretech, the company that developed our HIS, is under contract to maintain the HIS and has a team located at SNUH; this team could make timely improvements to the system in a straightforward manner. In addition, HealthConnect, a health care application development company, has been working on a mobile app with SNUH for a project to collect patient-reported outcomes and enable viewing of the data in the HIS. This familiarity with the hospital interfaces and data structures enabled rapid development and deployment of the LTSC app.

### Role of Health Care Information Exchange in the Pandemic Era

Health care information exchange between health care institutions plays an important role during pandemics and disasters [20-24]. In Korea, where the population has high accessibility to health care resources due to the national health insurance program, any patient can visit any hospital. Health records are fragmented among the health care institutions that the patient visits, as the EHR systems used by each institution are different and most are not interoperable [25]. To alleviate this problem, the Ministry of Health and Welfare initiated a nationwide healthcare information exchange (HIE) project in 2017. This service electronically and securely sends and receives medical records and image data of patients who have consented to provide personal information to medical institutions for patient care [26]. However, in the current HIE system, electronic information is generated only when the patient is referred to another medical institution; thus, it is difficult to use it in this situation. Furthermore, the project is not gaining traction as expected due to the cost burden to small and medium-sized hospitals to connect their EHRs with the national HIE system as well as the conflict between health care institutions and the government on reimbursement and penalty policies [27]. As most institutions were not prepared to share image data through the national HIE system, we developed a simple cloud-based image sharing platform to enable efficient exchange. This platform was immediately used for a 55-year-old woman who had no underlying disease and no symptoms at initial presentation but who progressed to pneumonia 3 days after admission. Her physician decided to transfer her to a local general hospital and shared her chest x-ray images through this system.

### Lessons From the MERS Outbreak and Application for COVID-19 in South Korea

South Korea learned important lessons from its 2015 Middle East respiratory syndrome (MERS) outbreak, including the importance of increased transparency, early detection, and rapid diagnosis [28-30]. The Korea Centers for Disease Control and the Health Insurance Review & Assessment Service developed the international traveler information system and provides the service to all health care institutions in Korea [31]. When the patient has visited countries that are being screened, the international traveler information system sends out a real time alert to allow staff to recognize and prevent possible exposure. Similarly, to minimize exposure, the system was used to inform the physician or nurse whether the patient was arriving from the epicenter of the COVID-19 epidemic in Korea, Daegu and Gyeongsangbuk Province, since February 18, 2020.

Although the current pandemic is ongoing, we should also learn from this experience. Telemedicine is ideal for proper triage, early diagnosis, isolation, and treatment of patients [32]. Even in the United States, where access to telemedicine services, coverage, and reimbursement is available, telemedicine use is relatively uncommon [33]. Telemedicine use is even rarer in Korea, which is one of the few countries where patient-facing telemedicine is explicitly banned [16]. All stakeholders involved, including the government, medical communities, civic groups, and politicians, have different opinions. Some believe that Korea should approach this issue differently from other countries due to the nature of the medical system in Korea, which has excellent accessibility and low costs [34]. However, to limit community transmission during the COVID-19 pandemic, the Ministry of Health and Welfare has temporarily approved the limited use of telemedicine with e-prescriptions [9]. With recent studies reporting the advantages of proactive use of IT systems and strategies in infectious patients [35-37], we hope that this experience provides an impetus for the stakeholders of the health care system to improve and expand on the use and application of ICT in health care.

### Conclusions

Faced with a novel infectious disease, we describe the implementation and experience of applying an ICT-based patient management system in the LTSC by SNUH. ICT-based tools and applications are increasingly gaining importance in health care, and we hope that our experience can provide insight into future technology-based infectious disease responses.

## Appendix

Multimedia Appendix 1 Supplemental Figure 1. Wearable vital sign recorder.

Multimedia Appendix 2 Supplemental Figure 2. Integration of data from the wearable vital sign device into the SNUH HIS.

Multimedia Appendix 3 Supplemental Figure 3. Central vital sign monitoring system.

Multimedia Appendix 4 Supplemental Table 1. Survey on perceived usefulness, perceived ease of use, and satisfaction with the mobile app. Supplemental Table 2. Survey on perceived usefulness, perceived ease of use, and satisfaction with wearable devices by patients in the LTSC.

## Abbreviations

CMS: central monitoring system
COVID-19: coronavirus disease
DICOM: Digital Imaging and Communications in Medicine
EHR: electronic health record
e-prescription: electronic prescription
HIE: healthcare information exchange
HIS: hospital information system
ICT: information and communications technology
IT: information technology
LTSC: living and treatment support center
MERS: Middle East respiratory syndrome
PACS: picture archiving and communication system
Q&A: question and answer
RT-PCR: real time polymerase–chain reaction
SNUH: Seoul National University Hospital
