# Supplementary figures and images for: Information Technology–Based Management of Clinically Healthy COVID-19 Patients: Lessons From a Living and Treatment Support Center Operated by Seoul National University Hospital
Source: J Med Internet Res. 2020 Jun 12;22(6):e19938. doi: 10.2196/19938 (PMC7294904; doi:10.2196/19938)

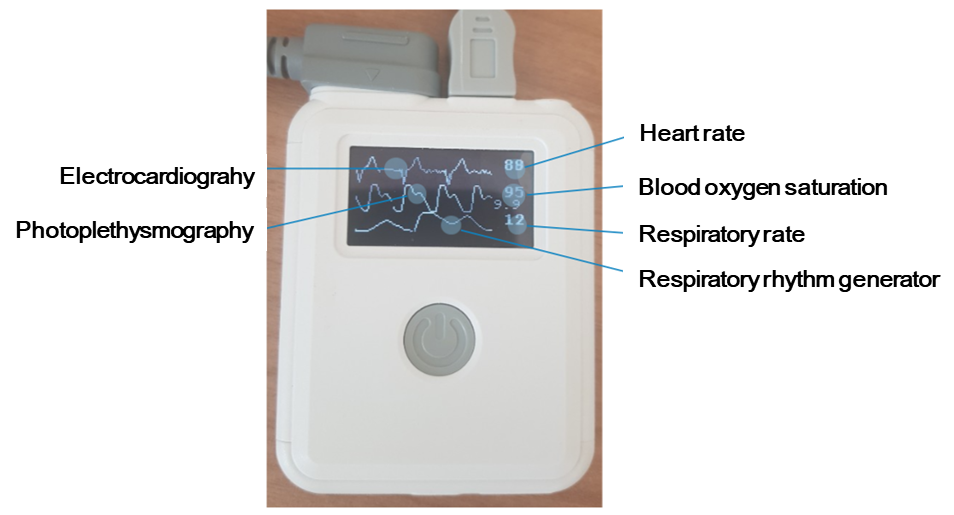

Supplement: Multimedia Appendix 1 [file jmir_v22i6e19938_app1.png]

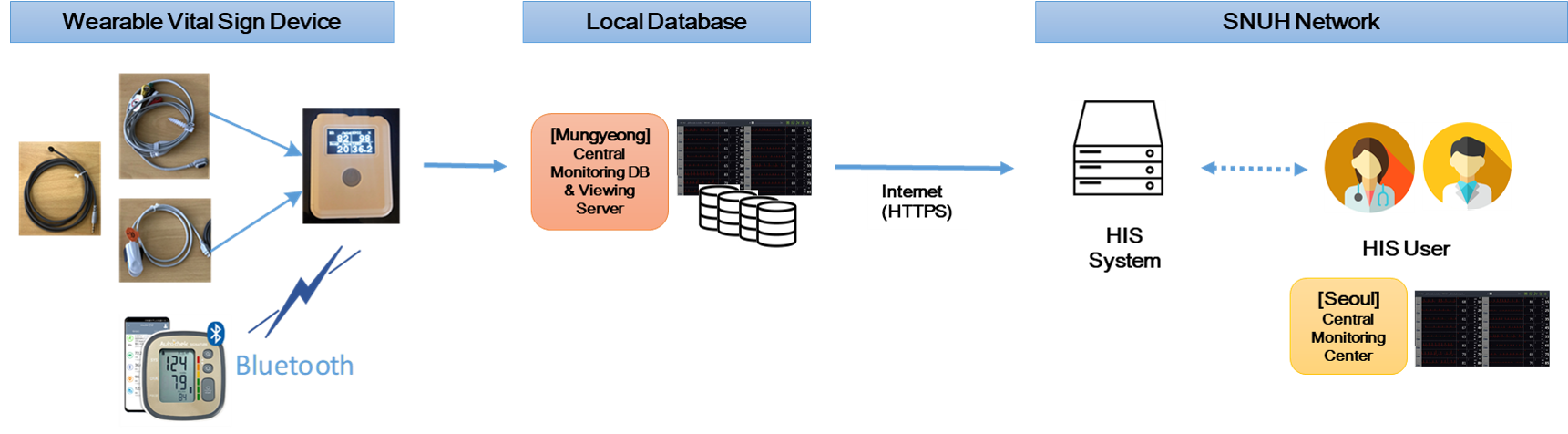

Supplement: Multimedia Appendix 2 [file jmir_v22i6e19938_app2.png]

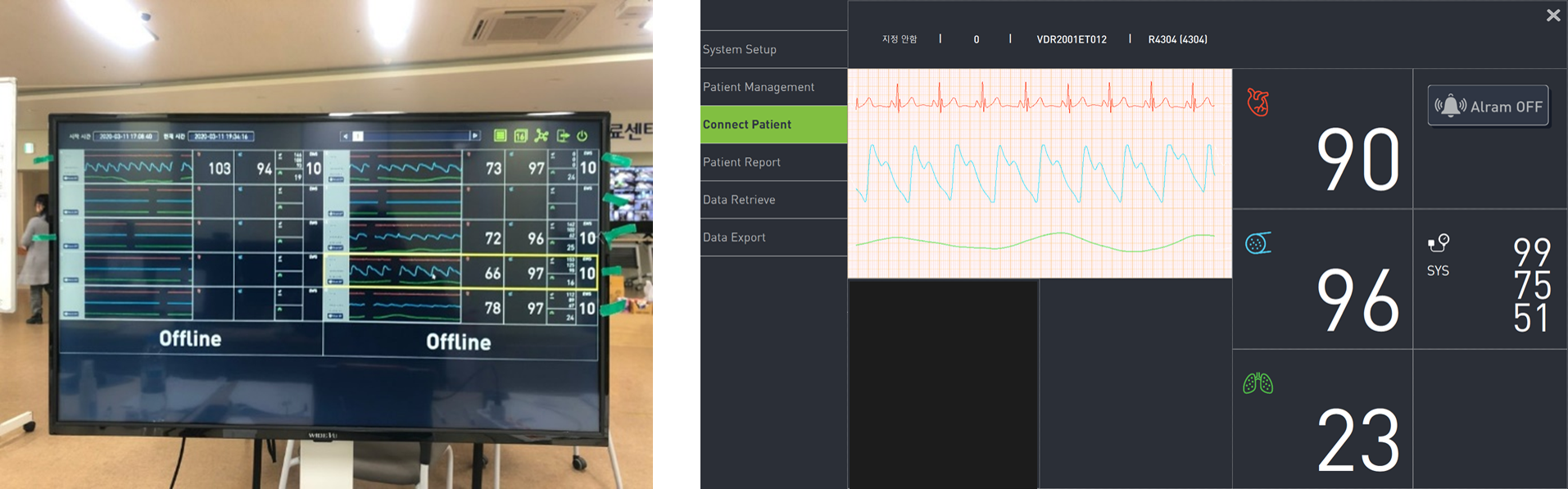

Supplement: Multimedia Appendix 3 [file jmir_v22i6e19938_app3.png]
