# Supplementary material for: Information Technology–Based Management of Clinically Healthy COVID-19 Patients: Lessons From a Living and Treatment Support Center Operated by Seoul National University Hospital
Source: J Med Internet Res. 2020 Jun 12;22(6):e19938. doi: 10.2196/19938 (PMC7294904; doi:10.2196/19938)
Supplement: Multimedia Appendix 4 [file jmir_v22i6e19938_app4.docx]

**Supplemental Table 1. Survey on Perceived Usefulness, Perceived Ease of Use and Satisfaction with the Mobile Application**

| **Categories** | **Mean** | **SD** |
| --- | --- | --- |
| **Patients** |  |  |
| Perceived usefulness | 4.62 | 0.48 |
| Satisfaction | 4.08 | 1.41 |
| Perceived ease of use | 3.81 | 0.41 |
| **Medical Staff** |  |  |
| Satisfaction | 4.10 | 0.64 |

**Supplemental Table 2. Survey on Perceived Usefulness, Perceived Ease of Use and Satisfaction with Wearable Devices by the Patients in the Living and Treatment Support Center**

| **Categories** | **Mean** | **SD** |
| --- | --- | --- |
| Perceived usefulness | 4.45 | 0.57 |
| Perceived ease of use | 4.30 | 0.59 |
| Satisfaction | 3.98 | 0.70 |
